# Waterborne Superhydrophobic and Superoleophobic Coatings for the Protection of Marble and Sandstone

**DOI:** 10.3390/ma11040585

**Published:** 2018-04-10

**Authors:** Dimitra Aslanidou, Ioannis Karapanagiotis, Dimitrios Lampakis

**Affiliations:** Department of Management and Conservation of Ecclesiastical Cultural Heritage Objects, University Ecclesiastical Academy of Thessaloniki, 54250 Thessaloniki, Greece; aslanidou.dimitra@gmail.com (D.A.); lampakis@teilar.gr (D.L.)

**Keywords:** superhydrophobic, superoleophobic, coating, cultural heritage, marble, sandstone

## Abstract

Silica nanoparticles were dispersed in an aqueous emulsion of alkoxy silanes and organic fluoropolymer. The dispersion was sprayed onto white marble and sandstone. The deposited composite coatings exhibited (i) superhydrophobicity and superoleophobicity, as evidenced by the high (>150°) static contact angles of water and oil drops as well as (ii) water and oil repellency according to the low (<7°) corresponding tilt contact angles. Apart from marble and sandstone, the coatings with extreme wetting properties were deposited onto concrete, silk, and paper, thus demonstrating the versatility of the method. The siloxane/fluoropolymer product was characterized using Fourier Transform Infrared Spectroscopy (FT-IR), Raman spectroscopy and Scanning Electron Microscopy equipped with an Energy Dispersive X-ray Spectrometer (SEM-EDX). Moreover, SEM and FT-IR were used to reveal the surface structures of the composite coatings and their transition from superhydrophobicity to superhydrophilicity which occurred after severe thermal treatment. The composite coatings slightly reduced the breathability of marble and sandstone and had practically no optical effect on the colour of the two stones. Moreover, the coatings offered good protection against water penetration by capillarity.

## 1. Introduction

Atmospheric water and rain can penetrate the porosity channels of natural stones causing direct (e.g., through freezing–thawing cycles) or indirect (e.g., by the deposition of pollutants) degradation effects in cultural heritage monuments, buildings and objects. The application of hydrophobic materials as protective coatings has been suggested as a potential solution for the surface protection of natural stones, used in cultural heritage [1,2,3,4,5]. More recently, advanced hybrid and composite materials of special surface structures were produced for stone protection, offering enhanced hydrophobicity and in some cases superhydrophobicity [6,7,8,9,10,11,12,13,14,15,16,17,18,19,20,21,22,23,24,25,26,27,28,29,30,31,32,33,34,35]. The static contact angle (*θ_S_*) of a water drop on a hydrophobic surface is 150° < *θ_S_* < 90°, whereas on a superhydrophobic surface, *θ_S_* becomes very large, typically > 150°. Consequently, superhydrophobic coatings can offer, in principle, better stone protection against the deteriorative activity of water, provided that superhydrophobicity is accompanied by water repellency. The hydrophobic/hydrophilic character of a surface is associated with the *θ_S_* but water repellency/adhesion is better assessed by the tilt contact angle (*θ_t_*), defined as the angle that a surface must be tilted to move a water drop. In a truly water repellent surface, *θ_t_* is very small, typically < 10°. Alternatively, instead of the *θ_t_*, the contact angle hysteresis, defined as the difference between the advancing and the receding contact angles, can be used to describe the dynamic wettability of a surface [7]. Large *θ_S_* (i.e., superhydrophobicity) is not necessarily accompanied by small *θ_t_* (i.e., water repellency). For example, a water drop on the surface of a rose petal corresponds to *θ_S_* > 150° [36,37]; yet, the drop cannot roll off even when the surface is turned upside down [36], implying that the drop is pinned [37] and adheres to the superhydrophobic surface of the rose petal. Consequently, both *θ_S_* and *θ_t_* are important to evaluate the protection efficacy which a coating offers to natural stone.

Several methods to produce superhydrophobic and water repellent coatings can be found in the literature [6,7,8,9,10,11,12,13,14,15,16,17,18,19,20,21,22,23,24,25,26,27,28,29,30,31,32,33,34,35]. For example, the wetting properties of polymer surfaces can change dramatically from a usual inherent hydrophobicity (or slight hydrophilicity) to superhydrophobicity and water repellency by embedding nanoparticles into the polymer matrices [6,7]. The presence of nanoparticles results in the formation of rough surface structures which induce extreme wetting properties [6,7]. This method of controlled nanoparticle-embedding into macromolecular matrices has some important advantages for the effective and sustainable protection of the cultural heritage [32]. It is an easy, low-cost, and one-step method which can be applied to treat large surfaces in ambient conditions and it uses common silane/siloxane products (or organic polymers), nanoparticles, and solvents. Silane/siloxane products are extensively used in stone consolidation and conservation [38]. Based on the aforementioned advantages, polysiloxane + nanoparticles (or organic polymer + nanoparticles) coatings have recently received significant attention and have been suggested for the protection of natural stone [6,7,8,9,11,12,13,14,16,17,20,22,23,24,25,27,28,29,32,33,34,35]. 

We have recently shown that, with careful selection of the silane/siloxane product and the concentration of added nanoparticles, the resulting polysiloxane + nanoparticles coating can evince both superhydrophobicity and superoleophobicity as well as water and oil repellency [39]. This is a major improvement, as monuments and other cultural heritage objects are often exposed to urban air pollution. Consequently, designing coatings which can repel not only water but also other liquids of lower surface tension (e.g., oil) is of great importance. To the best of our knowledge, this was the first report describing the production and deposition of a superoleophobic and oil repellent coating onto calcareous stones such as marble and sandstone [39]. In this previously published study, attention was focused largely on the interaction of the composite coatings with drops of various liquids and the measurements of the *θ_S_* and *θ_t_* contact angles [39]. In the present study, the wetting properties of the composite coatings are further investigated. Other important evaluation tests in the field of stone conservation, such as capillary water absorption, vapour permeability, and colour change, are carefully carried out. Furthermore, the selected macromolecular product (Silres BS29A) is characterized using several spectroscopic techniques. Spectroscopy is also used to explain a severe transition from superhydrophobicity to superhydrophilicity which is induced by extreme thermal treatment. Finally, it is shown that the coatings can be easily deposited on several substrates, thus demonstrating the versatility of the method.

## 2. Materials and Methods 

A commercially available emulsion of alkoxy silanes and organic fluoropolymer (Silres BS29A, Wacker, Munich, Germany) was diluted in distilled water to prepare a stock emulsion of 7 % *w*/*w*. Silica (SiO_2_) nanoparticles of 7 nm in mean diameter (Sigma-Aldrich, St. Louis, MO, USA) were added to the stock emulsion at a concentration of 2 % *w*/*w*. This concentration was selected after an extensive investigation on the effect of the SiO_2_ nanoparticle concentration on the wetting properties of the resulting composite film [39]. Dispersions were stirred vigorously for 30 min and sprayed immediately onto various substrates which were covered by composite films hereafter designated as Silres + nanoparticles coatings. Blocks of white marble and sandstone were used as substrates for extensive studies whereas pieces of silk and corrugated paper and blocks of concrete were used only for contact angle measurements. For comparison, pure Silres BS29A emulsion (without nanoparticles) was also sprayed onto the aforementioned substrates which were then covered by films hereafter designated as Silres coatings. 

Each coating was deposited by spraying 1 mL of the dispersion or emulsion while the tip of the nozzle (660 μm in diameter) of the airbrush system (Paasche Airbrush, Chicago, IL, USA) was held at a distance of 20 cm from the sample’s surface. Coated samples were annealed at 40 °C overnight to remove residual solvent (water) and kept at room temperature for two to three days. In another set of experiments, coated sandstone specimens were subjected to extreme thermal treatment; the specimens were heated at 750 °C for 3 s.

Drops of distilled water and olive oil were placed on the treated substrates. Static (*θ_S_*) and tilt (*θ_t_*) contact angles were measured using an optical tensiometer apparatus (Attension Theta, Gothenburg, Sweden). For the measurements of the *θ_t_*, the tilt rate was adjusted to 1°/s. The reported contact angles are averages of five measurements. Variations of the *θ_S_* and *θ_t_* measurements were within ±3° and ±1.5°, respectively.

The surface structures of the deposited coatings were revealed using Scanning Electron Microscopy (SEM; JEOL, JSM-6510, Tokyo, Japan). Prior to the SEM study, the samples were coated with a thin layer of carbon. Furthermore, SEM was coupled to an Energy Dispersive X-ray Spectrometer (EDX; Oxford Instruments, Abingdon, UK) and was used to characterize the Silres coating. The latter was further studied using Raman spectroscopy and Fourier Transform Infrared Spectroscopy (FT-IR).

Raman spectra were obtained at room temperature using a Horiba Lab RAM HR spectrometer (Kyoto, Japan) equipped with a Peltier-cooled charge coupled device and a microscope. Excitation was provided by the 514.5 nm line of an Ar+ laser with a laser power of ~1 mW focused on the sample by a standard 100× objective in a spot with a diameter of ~1 μm. Spectra were collected at 3 cm^−1^ resolution in the range of 155–3460 cm^−1^; typical accumulation times were 2–5 min. For the best performance of the spectrometer, the instrument was calibrated just before and right after each measurement by using the spectrum of a silicon wafer as a reference.

FT-IR measurements were obtained using a Spectrum Spotlight 400 PerkinElmer spectrometer (Waltham, MA, USA), coupled to a microscope fitted with both white light and infrared optics and a liquid nitrogen-cooled MCT (Mercury Cadmium Telluride) detector. Spectra were collected with a resolution of 4 cm^−1^ and were the result of 256 scans. The aperture size was 100 × 100 μm^2^. All spectra were processed following a two-step procedure consisting of baseline correction and normalization. Apart from the characterization of the Silres coating, the FT-IR spectroscopy was useful to reveal chemical changes which were induced on the product after extreme thermal treatment.

Colourimetric measurements were carried out using a Miniscan XE Plus spectrophotometer (HunterLab, Reston, VA, USA) and the results were evaluated using the L*, a* b* coordinates of the CIE 1976 scale. The reported results are averages of three measurements; variations are reported

The water capillary absorption measurements were carried out by the gravimetric sorption technique [5] using uncoated bare marble and sandstone blocks and stone specimens covered by Silres and Silres + nanoparticles coatings. Sample blocks (2.5 × 2.5 × 1 cm) were placed on a filter paper pad (1 cm of Whatman paper, No. 4) partially immersed in distilled water. The setup was within a climatic chamber (RH = 30% and T = 25 °C). Samples were removed and weighed for consecutive periods of treatment time to measure the amount of absorbed water. The measurements were carried out in triplicate and variations are provided as error bars.

For the vapour permeability tests, sample blocks were fixed on top of cylindrical poly(vinyl chloride) (PVC) containers which were partially (1/2) filled with water [5]. The sealed containers were placed in a climatic chamber, (RH = 20% and T = 30 °C). The containers were weighed every 24 h. Under constant vapour flow, the water vapour permeability was evaluated as the mass of water vapour passing through the surface unit in 24 h. Three consecutive measurements were carried out with intervals of 24 h and average values and variations were calculated.

## 3. Results and Discussion

### 3.1. Characterisation of Silres BS29A

**Figure 1:**
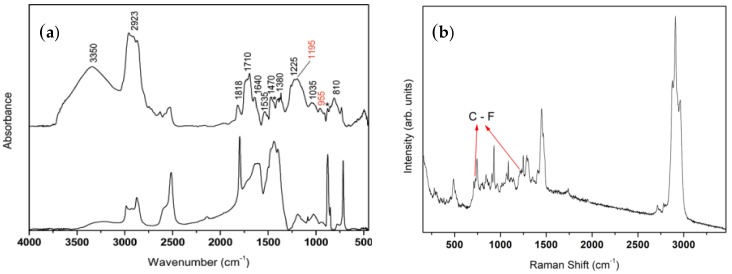
(**a**) FT-IR spectra of bare, uncoated marble (bottom) and marble coated by Silres (top). The peaks marked with an asterisk (*) are a result of the marble substrate. (**b**) Raman spectrum of Silres on marble.

Figure 1a shows the FT-IR spectrum of the Silres coating (Silres BS29A without nanoparticles) on marble. The spectrum of the uncoated marble is included in the figure for comparison. Observations including the coating spectrum of the hydroxyl group vibrations broad band at ~3350 cm^−1^, the dominant bands appearing at around 2900 cm^−1^ (which were assigned to the C–H modes of the methylene groups), the intense carbonyl band at ~1710 cm^–1^ and various C–O, C–H, Si–O, Si–C modes appearing in the 1650–700 cm^−1^ region, indicate that the coating consisted primarily of an alkoxy silane material [40,41]. Moreover, the characteristic absorption bands at 1195 cm^−1^ and 955 cm^−1^ were probably due to the C–F_2_ and C–F stretching modes, respectively [42], thus suggesting the presence of a fluoropolymer in the composition of the Silres coating. The FT-IR results (Figure 1a) are in agreement with the Raman spectrum which was obtained for the Silres coating, as shown in Figure 1b. The dominant bands appearing at around 2900 cm^−1^, which were assigned to the C–H modes of the methylene groups as well as the various C–O, C–H, Si–O, C–C, Si–C modes appearing in the low energy region (below 1500 cm^−1^), indicate that the Silres coating consists predominantly of an organo alkoxy silane [43]. Furthermore, the peak at 730 cm^−1^ may be attributed to symmetric stretching vibrations of C–F_2_ scissoring and the band at ~1214 cm^−1^ is probably a result of asymmetric stretching vibrations of C–F_2_ [44]. Consequently, the results of the FT-IR and Raman spectra in Figure 1 are in agreement with the product’s (Silres BS29A) description provided by the manufacturer (Wacker) and suggest that the Silres coating is a mixture of alkoxy silanes and fluoropolymer. The presence of silicon (Si) and fluorine F in the Silres composition was furthermore evidenced using SEM-EDX, as described in the Supplementary File. 

Fluoropolymer had a key role in the wetting properties of the coating as it resulted in a reduced surface energy and therefore enhanced the repulsive character of the coating against the deposition of any liquid. It was reported that the surface energy decreases in the order –CH_2_ > –CH_3_ > –CF_2_ > –CF_2_H > –CF_3_ [45]. Fluorinated and perfluorinated materials have low wettabilities and have therefore become the logical choice to produce superomniphobic materials, which are designed to repel any liquid [46]. However, fluorinated chemicals, including perfluoroalkylsilanes and fluoroacrylic polymers, have potentially effects on human health and on the environment [47]. Major attention has been focused on the hazardous properties of the short and long chain PFAAs (perfluoroalkyl acids) which have been recognized as contaminants of high concern owing to their high persistence, toxicity, bioaccumulation potential, and distribution in the environment [48,49].

### 3.2. Superhydrophobicity, Superoleophobicity, Water and Oil Repellency

Table 1 shows the *θ_S_* and *θ_t_* results of water and oil drops on Silres + nanoparticles coatings. For comparison, the corresponding results obtained on Silres coatings are included. Apart from marble and sandstone which were the target materials of the study, other materials including silk, corrugated paper, and concrete were coated. According to the results of Table 1, superhydrophobicity and water repellency were induced in all materials coated by Silres + nanoparticles, as water drops on these composite coatings corresponded to *θ_S_* > 150° and *θ_t_* < 10°. Likewise, superoleophobicity and oil repellency were achieved on Silres + nanoparticles coatings as oil drops on these composite coatings corresponded to *θ_S_* > 150° and *θ_t_* < 10°, except for treated paper where *θ_S_* = 145°. The results of Table 1 suggest that the use of SiO_2_ nanoparticles enhanced the hydrophobic and oleophobic character of the coatings and their repellency against both water and oil. Lower and higher *θ_S_* and *θ_t_*, respectively, of water and oil drops were measured on Silres than on Silres + nanoparticles coatings.

**Figure 2:**
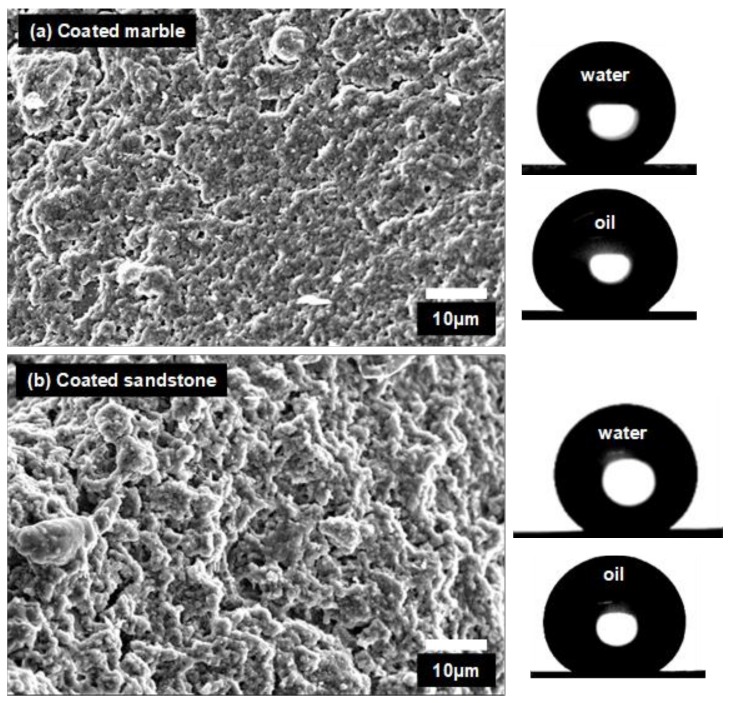
SEM images of (**a**) marble and (**b**) sandstone coated by Silres + nanoparticles films. Photographs of water and oil drops are included.

According to the results of Table 1, contact angles of water drops on Silres + nanoparticles coatings were nearly independent of the substrate material. Static contact angles (*θ_S_*) of water drops on the composite coatings were between a very small range from 158° to 165° and *θ_t_* values varied within 3°–5°. Larger variations of *θ_S_* and *θ_t_* are reported in Table 1 for water drops on Silres coatings. It was reported that the surface structure and the apparent wetting properties of a polysiloxane coating is affected by the roughness of the underlying substrate [7]. Accordingly, different *θ_S_* and *θ_t_* were measured on Silres coatings placed on different substrates (Table 1). However, when nanoparticles were added, their role in the surface structure of the composite coating was dominant and therefore any effect from the underlying substrate became negligible [7,32]. This effect of the nanoparticles was previously shown [7,32] and it is herein revealed in the SEM images of Figure 2. The surfaces of the Silres + nanoparticles coatings which were deposited on marble and sandstone are shown in Figure 2a,b, respectively. It is observed that the surface structures of the two coatings are similar and therefore practically independent of the substrate morphology. Consequently, the different underlying substrates should have no effect on the interaction of the two coated surfaces of Figure 2 with the water drops, as supported by the results of Table 1.

According to the results of Table 1, the *θ_S_* values of oil drops onto Silres + nanoparticles coatings on the different substrates were within 145°–160°. This is a much broader range compared to the 158°–165° range observed for the water drops. The surface tension of oil (=32 mN/m) is lower than that of water (=72 mN/m). Hence, oil drops are more sensitive in slight changes of the coating’s surface structure than water drops. This agrees with the results of a previously published paper [39]. It was reported that the effect of the nanoparticle concentration, which affected the resulting surface structure of the coating, was more dramatic on the *θ_S_* of oil drops compared to the *θ_S_* of water drops [39]. Consequently, small changes in the surface structure of the Silres + nanoparticles coatings induced by the underlying substrate had practically no impact on the shape of water drops but they have substantially affected the *θ_S_* of oil drops.

**Figure 3:**
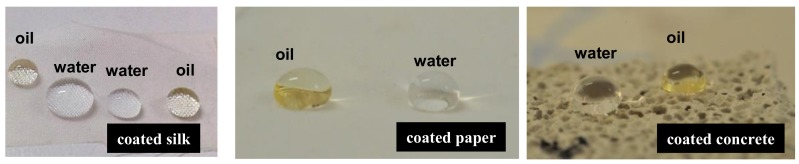
Water and oil drops on various materials which were coated by Silres + nanoparticles.

**Figure 4:**
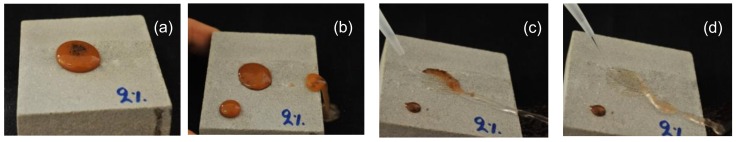
Demonstration of the easy/self-cleaning property of the Silres + nanoparticles coating on sandstone. Large drops of soiled water on the surface of coated sandstone (**a**,**b**) could be easily removed (**b**) and cleaned with fresh water (**c**,**d**).

Drops of water and oil on Silres + nanoparticles coatings which were deposited on silk, paper, and concrete are shown in Figure 3. The superhydrophobic and superoleophobic properties of the composite coatings are demonstrated. The easy/self-cleaning ability of the composite coatings is shown in Figure 4 using a block of coated sandstone. Large drops of water contaminated with soil were placed on the treated sandstone specimen (Figure 4a,b). The large drops rolled off when the specimen was slightly tilted (Figure 4b). The surface could be easily cleaned with fresh water which removed the soil contaminants (Figure 4c,d) without leaving any visible stain.

**Figure 5:**
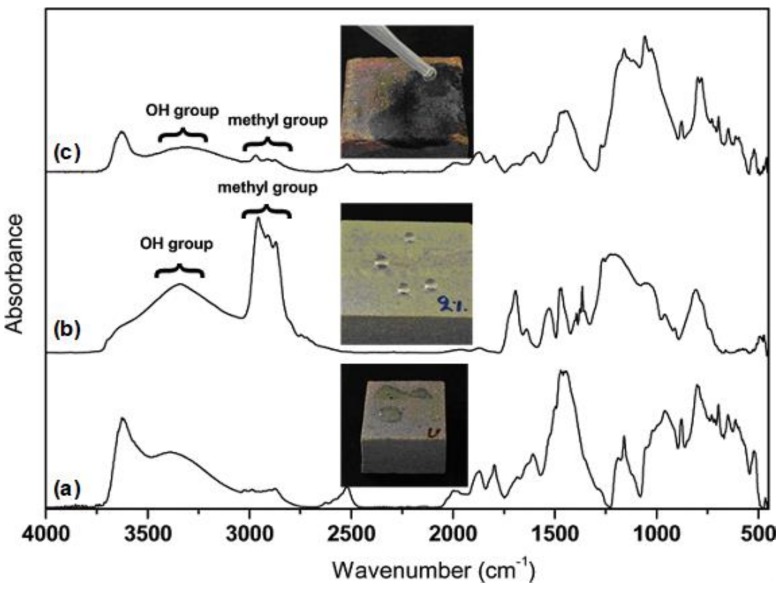
FT-IR spectra of (**a**) bare, uncoated sandstone, (**b**) fresh Silres + nanoparticles coating on sandstone and (**c**) Silres + nanoparticles coating on sandstone after extensive thermal treatment. Photographs which demonstrate the interaction of water drops with the three surfaces are included. In particular, the (**a**) hydrophilic, (**b**) superhydrophobic and (**c**) superhydrophilic properties of the corresponding surfaces are revealed.

An interesting property of polysiloxane surfaces is their transition from hydrophobicity/superhydrophobicity to hydrophilicity/superhydrophilicity, which occurs after thermal treatment at high temperatures. This is a result of the degradation of the hydrophobic functional groups and their replacement by hydrophilic functional groups generated through oxidation [50,51]. For the Silres + nanoparticles coatings, this transition is reported in Figure 5. In particular, Figure 5a shows the FT-IR spectrum of the bare sandstone which was taken as a reference background. The hydrophilic nature of the stone is revealed in the corresponding photograph of Figure 5a. The measurements in Figure 5b,c were taken from a Silres + nanoparticles coating on sandstone prior and after treatment at a high temperature (750 °C), respectively. The methyl group bands at ~2900 cm^−1^ appeared to be strong in the fresh composite coating (Figure 5b). However, the intensities of these C−H peaks were substantially reduced after thermal treatment whereas an intensity increment of the hydroxyl group broad band was recorded (Figure 5c). This chemical change in the coating’s surface led to a severe transition from superhydrophobicity to superhydrophilicity, as evidenced by the photographs included in Figure 5b,c. 

### 3.3. Water Capillary Absorption

**Figure 6:**
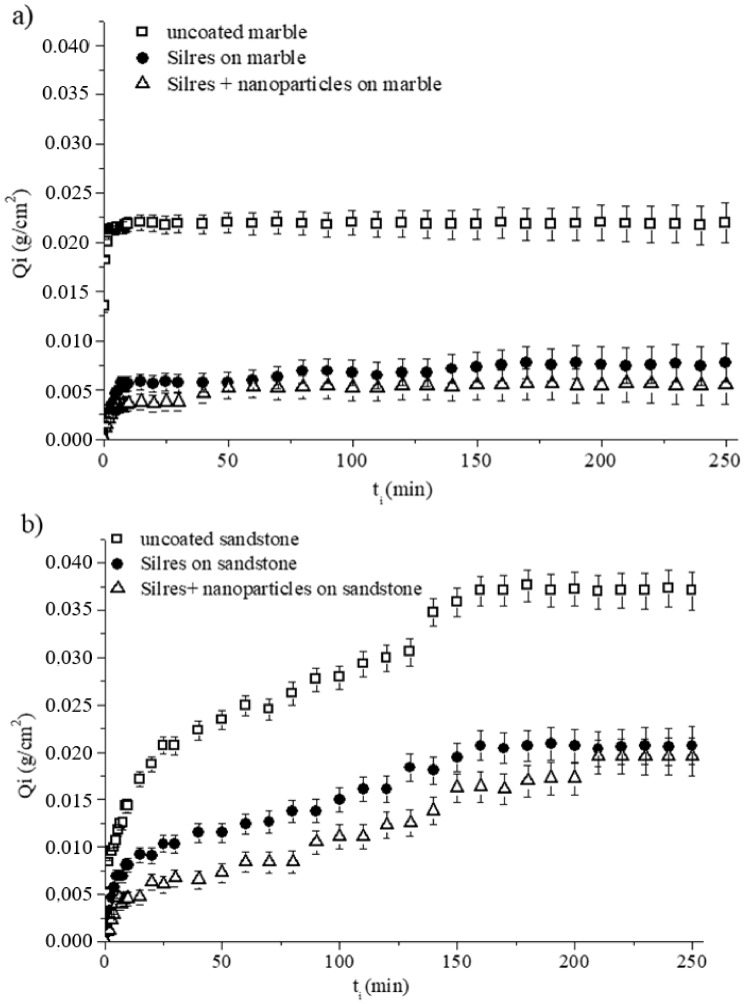
Amounts of absorbed water per unit area (Q_i_) as a function of treatment time t_i_, for (**a**) marble and (**b**) sandstone specimens which were uncoated and coated by Silres and Silres + nanoparticles.

The efficacy of the Silres + nanoparticles coatings to repel water absorbed by capillarity was evaluated for treated marble and sandstone specimens. For comparison, uncoated stone blocks and samples coated by Silres were included in the study. The amount of the absorbed water per unit area (Q_i_) after leaving the specimen in contact with water for time t_i_ was calculated as follows [52,53]:

(1) $$Q_{i}=\frac{w_{i}-w_{o}}{A}$$

where w_i_ is the weight of the sample after being in contact with water for time t_i_, w_o_ is the initial weight of the sample prior to the test and A is the sample’s area which had been in contact with water during the test. The calculated Q_i_ values were plotted as a function of time t_i_ for marble (Figure 6a) and sandstone (Figure 6b) specimens.

Figure 6a shows that the bare and coated marble specimens quickly became saturated in absorbed water, as evidenced by the recorded plateaus of the three Q_i_ − t_i_ curves. The amounts of water were absorbed according to the following order: uncoated sample -> sample coated by Silres -> sample coated by Silres + nanoparticles, with the latter being the sample that absorbed the least amount of water at each specific t_i_. Consequently, the use of nanoparticles in the coating had a positive effect in the protection of marble against water penetration by capillarity. However, according to the results of Figure 6a, the difference in the Q_i_ results between marble samples treated with hydrophobic Silres and superhydrophobic Silres + nanoparticles coatings, was within the experimental error bars.

The results for the sandstone specimens in Figure 6b qualitatively follow the same trend with the results of Figure 6a (marble). In particular, both Silres and Silres + nanoparticles coatings offered protection to the sandstone, against water absorption through capillarity, as their use resulted in reduced Q_i_ compared to the bare, uncoated sandstone sample (Figure 6b). Moreover, the superhydrophobic composite coating gave somewhat better results but its superiority over the hydrophobic Silres coating was within the experimental error (Figure 6b).

A comparison of the two Figure 6a,b, suggests that the saturation points were recorded in longer t_i_ for sandstone specimens which absorbed larger amounts of water compared to the corresponding marble specimens. In particular, saturations of the absorbed amounts of water were recorded at ~160 (Figure 6b) and ~10 min (Figure 6a) for sandstone and marble, respectively. Notably, after 250 min of contact with water, the amounts of water absorbed by the sandstone specimens were as follows: 0.037 g/cm^2^ for the uncoated sample, 0.021 g/cm^2^ for the sample coated by Silres, and 0.019 g/cm^2^ for the sample coated by Silres + nanoparticles (Figure 6b). The corresponding values for the marble specimens were lower at 0.022 g/cm^2^, 0.008 g/cm^2^, and 0.006 g/cm^2^, respectively (Figure 6a). Sandstone has a higher porosity and therefore a capacity to absorb larger amounts of water than marble. For this reason, the sandstone specimens needed a longer time to become saturated in absorbed water than the marble specimens, as revealed by the results of Figure 6.

For the maximum amounts of absorbed water corresponding to the plateaus of the curves in Figure 6, the reduction percentage of water absorption by capillarity (RC%) was calculated using Equation (2) [2,5]:

(2) $$RC\%=\left(\frac{m_{uw}-m_{tw}}{m_{uw}}\right)\times 100$$

where m_uw_ and m_tw_ are the masses of water absorbed by capillarity by the untreated/bare and treated/coated specimens, respectively. An ideal coating must eliminate the amount of water absorbed by capillarity (RC% = 100).

The results are summarised in Table 2. The application of the Silres coating resulted in reductions in the amounts of absorbed water by 66.5% and 44.7% for marble and sandstone samples, respectively. These values of RC% increased by roughly an additional 10%, when nanoparticles were added to the protective coatings. In particular, the RC became 75.1% and 53.8% for the marble and sandstone samples coated by Silers + nanoparticles.

### 3.4. Water Vapour Permeability

A coating that is designed for the protection of stone should not affect the water vapour transport properties. The effects of the Silres and the Silres + nanoparticles coatings on water vapour permeability were evaluated for the treated marble and sandstone specimens. The reduction percentage of vapour permeability (RVP%) was calculated according to Equation (3) [5]:

(3) $$RVP\%=\left(\frac{m_{uv}-m_{tv}}{m_{uv}}\right)\times 100$$

where m_uv_ and m_tv_ are the masses of water vapour penetrating the untreated/bare and treated/coated specimens, respectively. An ideal coating must have no effect on the water vapour permeability (RVP% = 0).

The RVP% results are reported in Table 2. The two coatings, Silres and Silres + nanoparticles, had roughly the same effect on the breathability of sandstone. A slight reduction of the RVP was noticed when nanoparticles were added to the protective coating, as the RVP% was reduced from 23.6 for sandstone coated by Silres to 20.0 for sandstone coated by Silres + nanoparticles. However, this was a very slight improvement on the transport of the water vapour. Sandstone has a high porosity which plays a dominant role in the vapour transport process. Therefore, the exact type of the surface protective coating had only a minor effect on the RVP% of the treated sandstone. This was demonstrated by the results reported for sandstone in Table 2 and is also supported by previously published reports. Rhodorsil 224 and Porosil VV Plus are two solvent-based siloxane products which were applied to sandstone and gave RVPs which were on the order of 20% [8]. Silica nanoparticles added to Rhodorsil and Porosil coatings did not have any major effect on the measured RVPs [8]. Consequently, three siloxane (Silres BS29A, Rhodorsil 224 and Porosil VV Plus) coatings on sandstone, with or without silica nanoparticles, gave roughly the same RVP, suggesting that the specific type of protective coating does not have any major effect on the breathability of sandstone.

In contrast to the highly porous sandstone, the use of nanoparticles had a major effect on the breathability of marble, which is a stone of low porosity. A major improvement on vapour transport was noticed when nanoparticles were added to the protective coating; the RVP% was reduced from 42.8 for marble coated by Silres to 16.8 for marble coated by Silres + nanoparticles. The augmented RVP% (=42.8), which was recorded when Silres was applied, indicates that the marble’s small pores were blocked by the protective material. The positive role that the nanoparticles had on water vapour permeability can be attributed to two effects. First, nanoparticles enhanced the coating’s surface roughness. Therefore, nanoparticles increased the active coating’s surface which was exposed to air; this facilitated the transport of vapour. Second, it has been reported that the diffusion rate of water vapour through a porous network increases as the hydrophobic character of the pores is enhanced [54]. In a previously published report, higher diffusion rates of water vapour through hydrophobic pores were measured than through hydrophilic pores [55]. In the present study, the nanoparticles enhanced the hydrophobic character of the coating inducing superhydrophobicity and therefore they should have a positive effect on water vapour transport through marble.

### 3.5. Colour Change

The optical effects of the Silres and Silres + nanoparticles coatings on marble and sandstone were evaluated through colourimetric measurements, as discussed below. The global colour differences (ΔΕ*) of marble and sandstone, induced upon coating application, was derived from Equation (4):

(4) $$\Delta E*=\sqrt{{\left(L_{t}^{*}-L_{u}^{*}\right)}^{2}+{\left(a_{t}^{*}-a_{u}^{*}\right)}^{2}+{\left(b_{t}^{*}-b_{u}^{*}\right)}^{2}}$$

where L*, a* and b* are the brightness, the red–green component and the yellow–blue component of the CIE 1976 scale, respectively. The “u” and “t” subscript characters correspond to the untreated/bare and treated/coated specimens, respectively.

The results are summarized in Table 3, which shows that the application of the superhydrophobic and superoleophobic Silres + nanoparticles coatings did not have any considerable optical effect on the aesthetic appearances of both marble and sandstone. The applications of the composite coatings led to colour changes in marble (ΔΕ* = 0.48) and sandstone (ΔΕ* = 1.43) which are not perceived by the human eye (ΔΕ* < 1.5).

According to the results of Table 3, the highest ΔΕ* (=3.11) was measured for sandstone coated by Silres. This optical effect is detectable by the human eye and it was largely a result of the change of the L* component which was reduced from 58.57 (uncoated sandstone) to 56.15 (sandstone coated by Silres). Moreover, a major contribution to the recorded ΔΕ* came from the change of the b* component which was increased from 3.43 to 5.37 when Silres was applied onto sandstone. The effect of the change of the a* component on the ΔΕ* was negligible. When SiO_2_ nanoparticles were embedded in the protective coating, their high L* (=88.00) and low b* (=−7.00) resulted in an increase of L* and decrease of b*, which became 58.00 and 4.74, respectively (sandstone coated by Silres + nanoparticles). Accordingly, this approached the corresponding values of the uncoated sandstone. Consequently, the use of the SiO_2_ nanoparticles had a positive effect on the optical change of sandstone: a lower ΔΕ* was measured when sandstone was coated with the composite coating than with Silres.

On the contrary, the use of the SiO_2_ nanoparticles had a negative effect on the optical change of marble: a higher ΔΕ* was measured when marble was coated with the composite coating compared to the colour change recorded when Silres was deposited onto the marble. In particular, ΔΕ* increased from 0.10 (marble coated by Silres) to 0.48 (marble coated by Silres + nanoparticles). In relative terms, this is a major increase. However, both colour changes are negligible in terms of perception by the human eye.

## 4. Conclusions

Superhydrophobic, water repellent, superoleophobic, and oil repellent properties were induced in marble and sandstone which were sprayed and coated with an aqueous dispersion that contained alkoxy silanes, organic fluoropolymer (revealed by FT-IR, Raman, and SEM-EDX studies) and silica nanoparticles. High static (>150°) and low tilt (<7°) contact angles of water and oil drops on coated stones were measured. Similar extreme wetting properties were achieved when the composite coatings were applied onto concrete, silk, and paper, demonstrating that the method can be effectively applied to treat various surfaces at ambient conditions. According to SEM images, the deposited coatings exhibited augmented roughnesses raised by surface structures at the micro/nano-meter scale. 

A severe transition of the coating wettability, from superhydrophobicity to superhydrophilicity, was observed upon extreme thermal treatment as hydrophobic (methyl) groups were replaced by hydrophilic (hydroxyl) groups according to a FT-IR study. 

The composite coatings slightly reduced the breathability of marble and sandstone by approximately 17 and 20% respectively, and had practically no optical effect on the colour of the two stones. Moreover, the coatings offered good protection against water penetration by capillarity.

The distinctive role of the silica nanoparticles in the aforementioned properties was elucidated, as coatings without nanoparticles were deposited on marble and sandstone and studied on a comparative basis with the composite coatings. Overall, it was demonstrated that the nanoparticles improved the properties of the protective coatings. 

## Figures and Tables

**Table 1:** Static (*θ_S_*) and tilt (*θ_t_*) contact angles of water and oil drops on Silres and Silres + nanoparticles coatings which were deposited on various substrate materials.

Substrate	Water Drops				Oil Drops			
	Silres		Silres + Nanoparticles		Silres		Silres + Nanoparticles	
	*θ_S_* (^o^)	*θ_t_* (^o^)	*θ_S_* (^o^)	*θ_t_* (^o^)	*θ_S_* (^o^)	*θ_t_* (^o^)	*θ_S_* (^o^)	*θ_t_* (^o^)
Marble	140	14	162	3	107	9	157	6
Sandstone	156	12	161	3	140	11	153	3
Silk	148	7	158	4	139	6	155	4
Paper	108	>90	165	5	109	>90	145	7
Concrete	156	4	161	3	149	4	160	3

**Table 2:** Results for the relative reduction of water absorption by capillarity (RC%) and vapour permeability (RVP%) for marble and sandstone specimens covered by Silres and Silres + nanoparticles coatings. Calculations were carried out using Equations (2) and (3).

Substrate	RC%		RVP%	
	Silres	Silres + Nanoparticles	Silres	Silres + Nanoparticles
Marble	66.5 ± 1.9	75.1 ± 0.6	42.8 ± 2.6	16.8 ± 1.5
Sandstone	44.7 ± 1.6	53.8 ± 5.4	23.6 ± 1.4	20.0 ± 1.0

**Table 3:** Mean values of colour coordinates of SiO_2_ nanoparticles, uncoated and coated marble and sandstone specimens. The global colour differences (ΔΕ*) of marble and sandstone, induced upon coating applications, are included.

Material	L*	a*	b*	ΔΕ*
SiO_2_ nanoparticles	88.00	−2.50	−7.00	-
Uncoated marble	94.60	−0.05	2.03	-
Marble coated by Silres	94.65	0.01	2.10	0.10 ± 0.01
Marble coated by Silres + nanoparticles	95.05	−0.02	1.86	0.48 ± 0.02
Uncoated sandstone	58.57	−0.35	3.43	-
Sandstone coated by Silres	56.15	−0.19	5.37	3.11 ± 1.30
Sandstone coated by Silres + nanoparticles	58.00	−0.39	4.74	1.43 ± 0.30

## Supplementary Materials

Supplementary File 1
