# Supplementary material for: Waterborne Superhydrophobic and Superoleophobic Coatings for the Protection of Marble and Sandstone
Source: Materials (Basel). 2018 Apr 10;11(4):585. doi: 10.3390/ma11040585 (PMC5951469; doi:10.3390/ma11040585)
Supplement: Supplementary File 1 [file materials-11-00585-s001.pdf]

Supplementary File

Waterborne superhydrophobic and superoleophobic coatings for the protection of marble and sandstone

Dimitra Aslanidou[1] and Ioannis Karapanagiotis[1,*], Dimitrios Lampakis[1]

[1] University Ecclesiastical Academy of Thessaloniki, Department of Management and Conservation of Ecclesiastical Cultural Heritage Objects, Thessaloniki 54250, Greece; aslanidou.dimitra@gmail.com, lampakis@teilar.gr

* Correspondence: y.karapanagiotis@aeath.gr; Tel.: +30-2310-301-784

Scanning electron microscopy was coupled to an Energy Dispersive X-ray Spectrometer (SEM-EDX) and was used to characterize the Silres coating which was deposited on marble. The SEM-EDX results are shown in Figure 1S. The detections of Si and F in the spectrum of Figures 1S are attributed to the siloxane and fluoropolymer, respectively, which are contained in the Silres coating (Silres BS29A). Other elements included in the spectrum such as Ca, Al and Mg originate from the marble substrate.

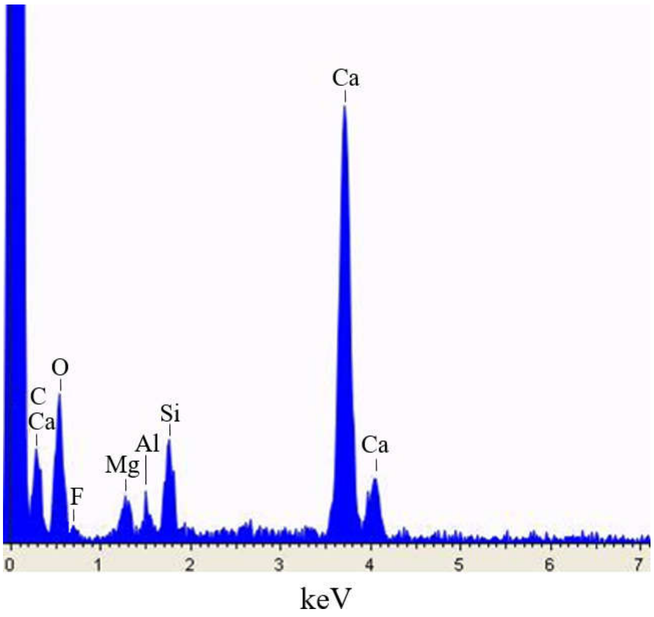

Figure 1S. SEM-EDX spectrum of Silres coating on marble.
